# Editorial: Unraveling GI cancer heterogeneity through single-cell multi-omics approaches

**DOI:** 10.3389/fgene.2026.1869790

**Published:** 2026-05-20

**Authors:** Rick J. Jansen, Roberta Bardini, Elena Grassi

**Affiliations:** 1 Biostatistics Core, Masonic Cancer Center, University of Minnesota Twin Cities, Minneapolis, MN, United States; 2 Department of Control and Computer Engineering, Politecnico di Torino, Turin, Italy; 3 Candiolo Cancer Institute, FPO-IRCCS, Candiolo, Torino, Italy; 4 Department of Oncology, University of Torino, Candiolo, Torino, Italy

**Keywords:** GI cancers, multi-omics, RNA, single cell, spatial transcriptomics

## Introduction

Gastrointestinal (GI) cancers represent a significant global health burden, accounting for 26% of cancer incidence and 35% of all cancer-related deaths (Arnold et al., 2020). While the primary sites—colorectum, stomach, liver, esophagus, and pancreas—are functionally diverse, they share a common epithelial origin and are linked within the same physiological system. The challenge in treating these malignancies lies in their profound genetic and phenotypic heterogeneity, as well as the complex interplay between malignant cells and their surrounding tumor microenvironment (TME) (Zhang et al., 2022). In addition there has been a recent worldwide surge in incidence in younger individuals, with still unknown etiology (Xia et al., 2025).

This Research Topic highlights the transformative power of single-cell technologies and spatial transcriptomics in decoding these complexities. By capturing a variety of genomic data, the collected research offers an integrated view of the mechanisms driving tumor initiation, progression, and therapeutic response. It is important to note the typical limitations of these types of studies which include small sample sizes and a need to validate published results.

## Decoding the tumor microenvironment

A recurring theme across this Research Topic is the detailed characterization of the TME, particularly immune cell dynamics that help us gain understanding of the variability in patient treatment response and outcomes:

- Pancreatic Ductal Adenocarcinoma (PDAC): Wang et al. identified a novel “benign” M2-like tumor-associated macrophage (TAM) subtype. Unlike malignant TAMs (they call mM2-like TAMs) that promote progression via MIF and SPP1 secretion, these bM2-like TAMs facilitate T cell activation, favorable stroma modeling via ɑSMA+ myofibroblasts, and result in better overall survival.
- Hepatocellular Carcinoma (HCC): Using ultra-depth spatial sequencing, Yu et al. revealed that HCC cells exhibit significantly more heterogeneity and more intense and broader responses to nearby immune cells compared to benign tissue. They observed that immune cells appear to selectively deactivate cancer signaling pathways while leaving similar pathways unaffected in normal liver tissue. HCC cells appear to use immune evasive mechanisms like reducing ligand-receptor communications and immune regulatory signaling pathways.
- Liver Fibrosis: Qiao et al. integrated single-cell and spatial data to identify endothelial cells as key fibrosis-associated clusters, pinpointing three prognostic genes (*LUC7L3*, *CREB1*, and *YIPF4*) via a random survival forest algorithm that influence survival and immune infiltration. These key genes were correlated with increased M0 macrophages, exhausted helper T-cells, and were suggested by bioinformatics modeling, oncoPredict, to influence chemotherapy response. These authors took the critical step of confirming the pro-cancer role of YIPF4 using laboratory assays; observing increased expression associated with increased proliferation, migration, and invasion.

## Insights into progression and metastasis

Understanding how tumors evolve from precancerous states to metastatic disease is critical for early intervention:

- Colorectal Morphogenesis: Gong et al. tracked the trajectory of granular-type laterally spreading tumors (LST-Gs). They found that metaplastic differentiation and upregulated Arp2/3 complex expression facilitate cell migration along the basement membrane, mirroring the transition to carcinoma. Remodeling of the TME occurred in areas of LST-Gs increasing immune suppression and myeloid interaction. Another key observation was the upregulation of ribosome biogenesis and metabolic pathways highlighting the increasing energy demands of the spreading tumors.
- Rare Neoplasms: Ayala et al. provided a rare look at appendiceal mucinous neoplasms (AMN) and their progression to pseudomyxoma peritonei (PMP). Their work identified distinct metastatic signatures (Goblet-like, Enterocyte-like, and tumor-associated cells) involving lipid metabolism and JAK-STAT signaling, as well as significant clonal heterogeneity within single patients. The presence of mucin provides both a physical barrier protection from immune cells and glycan structural barrier protection from cell death. As tumor cells were driven to metastasis, they increased their EMT markers and upregulated their nutrient-poor metabolism pathways like oxidative phosphorylation and cholesterol. The combination of spatial and single-cell data showed that metastatic lesions had high levels of exhausted T-cells and myeloid infiltration leading to reduced immune responses.

## Clinical implications and heterogeneity

The Research Topic also addresses the practicalities of precision medicine. Jansen et al. explored intratumor heterogeneity by comparing paired samples from the same patient. They attempt to address the question, “Are we able to create reliable treatment plans for a patient based on RNA-seq collected from one sample or is there too much variation across samples to do so accurately?” Their finding that cell type proportions—particularly NK cells and macrophages—vary significantly between specimens suggests that a single biopsy may be insufficient for effective treatment planning.

## Conclusion

The studies presented here demonstrate that the integration of single-cell RNA-seq, spatial transcriptomics, and multi-omic approaches is essential for unraveling the biological processes of GI cancers. By identifying cell-specific mechanisms and recurring patterns across tumor types and samples, this research provides a foundation for identifying new therapeutic targets and improving prognosis prediction in some of the world’s most lethal malignancies.
