# Correction: Editorial: Unraveling GI cancer heterogeneity through single-cell multi-omics approaches

**DOI:** 10.3389/fgene.2026.1907386

**Published:** 2026-06-25

**Authors:** Rick J. Jansen, Roberta Bardini, Elena Grassi

**Affiliations:** 1 Biostatistics Core, Masonic Cancer Center, University of Minnesota Twin Cities, Minneapolis, MN, United States; 2 Department of Control and Computer Engineering, Politecnico di Torino, Turin, Italy; 3 Candiolo Cancer Institute, FPO-IRCCS, Candiolo, Torino, Italy; 4 Department of Oncology, University of Torino, Candiolo, Torino, Italy

**Keywords:** GI cancers, multi-omics, RNA, single cell, spatial transcriptomics

Affiliation “Candiolo Cancer Institute, FPO-IRCCS, Candiolo (TO), Italy” was omitted from the paper. This affiliation has now been added for author Elena Grassi as affiliation 3. The previous affiliation 3 “Department of Oncology, University of Torino, Candiolo, Torino, Italy” now becomes affiliation 4.

The original article has been updated.

